# First-Principles Calculations of the Electronic Structure and Optical Properties of Yttrium-Doped ZnO Monolayer with Vacancy

**DOI:** 10.3390/ma13030724

**Published:** 2020-02-05

**Authors:** Qian Wu, Ping Wang, Yan Liu, Han Yang, Jingsi Cheng, Lixin Guo, Yintang Yang, Zhiyong Zhang

**Affiliations:** 1 State Key Laboratory of Integrated Services Networks, School of Telecommunications Engineering, Xidian University, Xi’an 710071, China; 2 School of Physics and Optoelectronic Engineering, Xidian University, Xi’an 710071, China; 3 School of Microelectronics, Xidian University, Xi’an 710071, China; 4 School of Information Science & Technology, Northwest University, Xi’an 710127, China

**Keywords:** ZnO monolayer, yttrium, vacancy, electronic structure, optical properties, first-principles calculations

## Abstract

The electronic structures and optical characteristics of yttrium (Y)-doped ZnO monolayers (MLs) with vacancy (zinc vacancy, oxygen vacancy) were investigated by the first-principles density functional theory. Calculations were performed with the GGA+U (generalized gradient approximation plus U) approach, which can accurately estimate the energy of strong correlation semiconductors. The results show that the formation energy values of Y-doped ZnO MLs with zinc or oxygen vacancy (V_Zn_, V_O_) are positive, implying that the systems are unstable. The bandgap of Y-V_Zn_-ZnO was 3.23 eV, whereas that of Y-V_O_-ZnO was 2.24 eV, which are smaller than the bandgaps of pure ZnO ML and Y-doped ZnO MLs with or without V_O_. Impurity levels appeared in the forbidden band of ZnO MLs with Y and vacancy. Furthermore, Y-V_Zn_-ZnO will result in a red-shift of the absorption edge. Compared with the pure ZnO ML, ZnO MLs with one defect (Y, V_Zn_ or V_O_), and Y-V_Zn_-ZnO, the absorption coefficient of Y-V_O_-ZnO was significantly enhanced in the visible light region. These findings demonstrate that Y-V_O_-ZnO would have great application potential in photocatalysis.

## 1. Introduction

Since the third scientific and technological revolution, energy has become essential for economic development. At present, fossil energy, such as oil, natural gas, and coal, is the most consumed energy in the world. However, the energy resources on earth are limited, and these fossil fuels will rapidly run out in the first half of the 21st century. Therefore, the vigorous development of renewable energy is not only conducive for sustainable economic development but also for alleviating the increasingly serious energy and environmental problems. As a potential renewable energy source, solar energy, especially visible light (which accounts for most of the solar energy), is believed to contribute to mitigating these problems through photocatalytic technology (also known as semiconductor-based photocatalytic technology) [1]. Semiconductor-based photocatalysis has attracted considerable attention due to its direct conversion of solar energy to easily stored hydrogen, as well as free secondary pollution [2,3]. The basic principle of semiconductor-based photocatalytic technology is the energy band theory of solids. Under visible light irradiation, the photons can be adsorbed to generate electrons (e^-^) in the conduction band (CB) and holes (h^+^) in the valence band (VB) once their energy is greater than the bandgap energy (E_g_) of the semiconductor photocatalyst. These photo-generated charge carriers will react with surface molecules (such as H_2_O, adsorbed O_2_) to undergo secondary reactions to produce radical species (OH, $O_{2}^{-}$), which further react with the organic compounds and reduce them to harmless products [4,5]. Since the water splitting phenomenon with titanium dioxide (TiO_2_) and platinum as electrodes was discovered in 1972, semiconductor photocatalysts, such as TiO_2_, CdO, and ZnO, have been widely investigated due to their excellent electronic and optical properties, non-toxicity, and low cost [6,7,8]. As a kind of II-VI compound semiconductor, ZnO can also serve as a high-activity semiconductor photocatalyst because it has high chemical stability, high carrier mobility, large exciton binding energy (60 meV), mature synthesis technology, and tunable properties [9,10,11]. However, the wide bandgap (3.37 eV) of ZnO will result in low absorbability (4%) under sunlight irradiation [12], which is not conducive to the photocatalytic utilization of ZnO in the visible light region. To enhance the photocatalytic efficiency, various synthesis methods and surface modifications (doping, composite, and deposition) have been adopted to change the crystal structure, size, morphology, and bandgap [13]. Metal doping can effectively promote the visible light harvesting ability of catalysts [14,15,16,17,18,19,20]. Yttrium (Y) is an environmentally-friendly and abundant rare earth (RE) element [21]. The radius of yttrium is close to the atomic radius of zinc (Zn), which facilitates the insertion of Y ions into the ZnO lattice [22]. So far, some studies on Y-doped ZnO have shown that impurity levels could be introduced in the forbidden band by Y doping, and the valence electron transition with low-energy excitation could improve the utilization of visible light [23,24,25,26]. The authors in [21,27] observed that the optical transmittance of Y-doped ZnO films in the visible spectral range diminishes with yttrium doping, suggesting that the absorption coefficient increases in the visible light region.

During preparation, ZnO will exhibit intrinsic defects, such as zinc vacancy (V_Zn_) and oxygen vacancy (V_O_), and the role of these vacancy defects in the optical properties of ZnO has been explored experimentally and theoretically [28,29,30]. Tang et al. [28] found that V_O_ narrowed the bandgap of ZnO, and the results of photocatalytic experiments reflected that V_O_ can not only act as impurity levels in the band structure of ZnO but also function as electron traps to accept the photo-generated electrons. Wang et al. [29] prepared pristine ZnO films, and found that the introduction of the green luminescence is correlated with the formation of the Zn vacancy-related defect (V_Zn_). Theoretical studies of the effect of vacancy defects in metal element-doped ZnO were performed in [31,32,33,34,35,36]. Bai et al. [31] studied the electronic and optical properties of 2D ZnO:Mg/Be with V_O_ or V_Zn_. The results indicated that V_O_ will cause a blue-shift, whereas V_Zn_ will cause a red-shift in the optical absorption spectra. Alessandra et al. [32] presented a first-principles study on the effect of native point defects in Al:ZnO transparent conductive oxide. They found that V_O_ defects maintain the electrical properties but worsen the transparency of native Al:ZnO, whereas V_Zn_ defects are strong electron acceptors that can destroy the metal-like conductivity of the system. Meng et al. [33] studied the formation energies, electronic, and optical properties of pure ZnO and Er-doped ZnO with and without incorporating the intrinsic point defects (IPDs). The strong interactions between the Er dopant and acceptor-type IPDs in ZnO were perceived as one of the important reasons for the strong light absorption in the visible and infrared regions. For the yttrium element, Li et al. [36] calculated the electronic and ferromagnetic properties of a Y-doped ZnO (0001)-Zn polar surface with and without point defects. The calculated results showed that V_Zn_ is an acceptor that can trap electrons to balance the electrovalence, and V_O_ can introduce an impurity level in the bandgap near the Fermi level.

Until now, most studies on yttrium element-doped ZnO have focused on bulk, surface, and thin film forms [21,23,36]. Compared with these forms, ZnO ML of two-dimensional (2D) material has unique properties, such as larger surface areas, furnishing photocatalytic reaction sites, and a shorter transportation path for the photo-induced carriers, which are beneficial to promote photocatalytic performance [37,38]. Freeman et al. [39,40] firstly predicted the two-dimensional layered phase of ZnO, and they found that the 2D structure of ZnO prefers a graphitic-like structure when the number of ZnO (0001) layers is reduced, due to the depolarization of the surface. Experimentally, Deng et al. [41] synthesized a ZnO ML by reactive deposition of Zn on Au (111), which confirmed the existence of a ZnO ML graphite-like structure. Tan et al. [42] investigated the structural, electronic, and magnetic properties of RE metals (RE = Y, Ce, Eu, Gd, and Dy) doped ZnO ML using the DMol^3^ package. The ZnO ML with Ce, Eu, Gd, or Dy was magnetic, but Y-doped ZnO ML was a nonmagnetic semiconductor. ZnO ML undergoes a transition from semiconductor to metal with the presence of Y doping. Although the electronic and optical properties of ZnO ML with yttrium (Y) or vacancies have been described, the structural and optical properties of yttrium-doped ZnO ML with vacancy defect (V_Zn_ or V_O_) have not been investigated yet.

In this work, the first-principles calculations of yttrium (Y)-doped ZnO ML with zinc vacancy (V_Zn_) or oxygen vacancy (V_O_) are performed. For comparison, pristine ZnO ML and ZnO MLs with Y or vacancy are also calculated. The crystal structures and formation energies of pure ZnO ML and ZnO ML with Y or vacancy are studied to ensure the rationality of the models. The electronic structures and optical properties of the ZnO ML doping systems are obtained, analyzed, and compared. This work also analyzes the possible photocatalytic reaction mechanism to further understand the photocatalytic principle of Y doping with vacancy on ZnO MLs.

## 2. Calculation Method 

First-principles calculations were performed on the basis of the density functional theory (DFT) [43]. Compared with LDA (local density approximation) functionals, GGA (generalized gradient approximation) functionals (including PW91 [Perdew-Wang 91], PBE [Perdew-Burke-Ernzerhof] and RPBE [Revised-Perdew-Burke-Ernzerhof]) have more precise energy and structure and are more suitable for open systems. PW91 was first proposed by Perdew et al. in 1991 [44]. PBE was first proposed by Perdew and Burke in 1996. Improvements over PW91 include an accurate description of the linear response of the uniform electron gas, correct behavior under uniform scaling, and a smoother potential [45]. RPBE was first proposed in 1998 with improvements in the chemisorption energy of atoms and molecules [46]. Therefore, we adopted the GGA with PBE function as the correlation function [47], and the exchange-correlation interaction between ions and electrons was described by ultrasoft pseudopotentials [48]. The valence electron orbitals for Zn, O, and Y were chosen as Zn 3d^10^4s^2^, O 2s^2^2p^4^, and Y 4d^1^5s^2^, respectively. In this work, all the calculations of structural stability, electronic, and optical properties of ZnO ML with and without Y or vacancy were performed by CASTEP (Cambridge Sequential Total Energy Package). Geometry optimization was performed with all atoms moving freely and cells optimized fully. In order to investigate the impact of Y and vacancy doping concentration on the stability of ZnO ML, models of 2 × 2 × 1, 3 × 3 × 1, and 4 × 4 × 1 supercells were considered. The co-existence of Y and vacancy in ZnO ML was investigated in the most stable doping models. The plane-wave cutoff energy was 400 eV, the Monkhorst–Pack grid of k-point sampling was set to 4 × 4 × 2 in the Brillouin zone (BZ) [49]. In the self-consistent field calculation, the convergence precision of the energy charge was 1.0 × 10^−5^ eV/atom, the maximum displacement convergence criterion was 1.0 × 10^−3^ Å, the maximum force acting on each atom was 0.03 eV/Å, and the maximum stress was 0.05 GPa. A vacuum region of 15 Å was applied along the z-direction above the ZnO ML to avoid the interactions caused by periodic boundary conditions. The intrinsic coordinates and crystal parameters were completely relaxed in the geometric optimization. The GGA+U method was adopted to optimize the energy of systems and correct the bandgap value of ZnO ML [50]. The Hubbard parameters U_d,Zn_ of 10 eV [51], U_p,O_ of 7 eV [51], and U_d,Y_ of 2 eV [23] were employed. Besides this, all the calculations were performed under spin polarization and the same U parameters. 

## 3. Results and Discussion

### 3.1. Crystal Structure and Structural Stability

**Figure 1:**
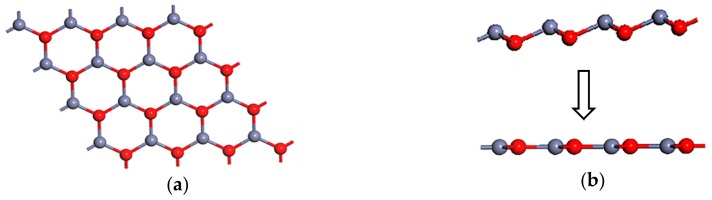
Graphene-like ZnO ML with 4 × 4 × 1 supercell. (**a**) The top view, (**b**) the side view of the original and the optimized structure.

To better study the properties of the ZnO ML with Y or vacancy, the crystal structure and stability of the ZnO ML should be investigated first. ZnO ML was acquired by cutting the bulk wurtzite ZnO with (0001) polar surface. The top view of the optimized ZnO ML is shown in Figure 1a, which illustrates a graphene-like structure. Each atom in the ZnO ML was three-fold coordinated, which is different from the four-fold coordinate Zn and O atoms in the bulk wurtzite structure [52]. Figure 1b shows the side view of the original and optimized ZnO MLs. The side view of the original ZnO ML was a ripple surface [53] because it retained the wurtzite structure of bulk ZnO (α = 90°, β = 90°, γ = 120°). However, the side view of the optimized ZnO ML became flat, the O-Zn-O bond angle was 120°, and the bond length between Zn and O atoms was 1.895 Å, which agrees well with the previous theoretical values [54,55]. The Zn-O bond length in the ZnO ML was smaller than the corresponding bond length in bulk ZnO (2.01 Å). This discrepancy is due to the stronger sp^2^ orbital hybridization of ZnO in the 2D structure than the sp^3^ orbital hybridization in bulk ZnO [43].

To assess the stability of the ZnO ML with and without Y or vacancy, the formation energies of the ZnO MLs were calculated. The formation energy (*E_f_*) can be calculated by the following [56]:

(1) $$E_{f}=E_{defect}-E_{perfect}-\sum_{i}n_{i}\mu_{i}.$$

Here, *E_defect_* is the total energy of ZnO ML with defect (Y, V_Zn_, V_O_), *E_perfect_* is the total energy of the pristine ZnO ML without any impurity or vacancy, *n_i_* is the number of atoms of type *i* added to (*n_i_* > 0) or removed from (*n_i_* < 0) the initial system, and *μ_i_* is the chemical potential of an atom of type *i* [15]. The *μ_i_* is the chemical potential obtained from the energy of one zinc or yttrium atom in a large 2D supercell (i.e., μ_Y_ = −188.521 eV, μ_Zn_ = −1708.148 eV, μ_O_ = −429.571 eV). 

**Figure 2:**
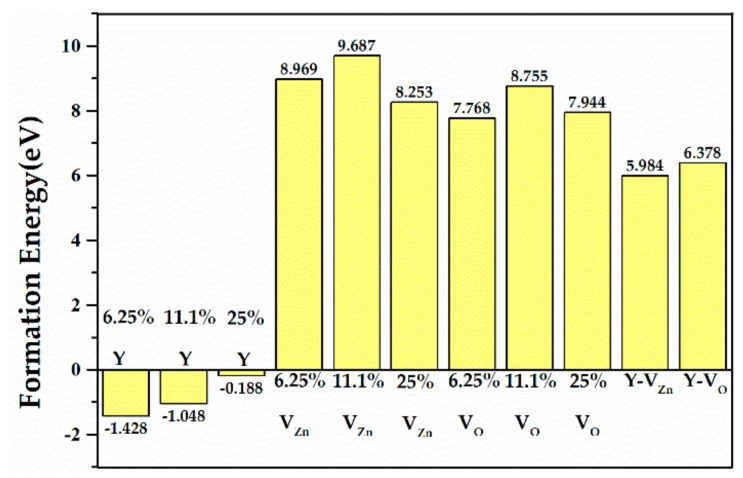
The formation energies of ZnO MLs with Y, V_Zn_, V_O_, and ZnO MLs with Y and vacancy.

**Figure 3:**
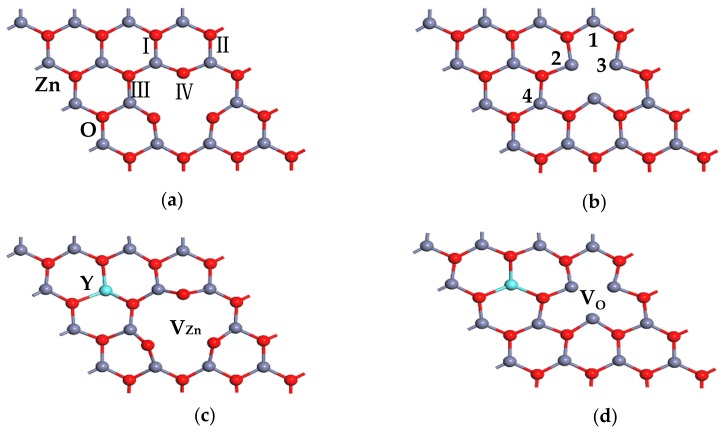
The optimized ZnO MLs with (**a**) V_Zn_, (**b**) V_O_, (**c**) Y and V_Zn_, (**d**) Y and V_O_.

Figure 2 shows the formation energies of ZnO MLs with and without Y or vacancy. As shown in the figure, all formation energy values of Y single-doped ZnO ML were negative, indicating that Y-doped ZnO ML can spontaneously form in the experiment. The formation energy value of Y single-doped ZnO ML changed from −1.428 eV to −0.188 eV with increasing Y concentration. Hence, ZnO ML can be inferred to be the most stable system when the doping concentration of Y is 6.25 at.%. Besides this, the formation energy of V_Zn_-ZnO was close to that of V_O_-ZnO, which were both positive. This finding indicates that the systems are unstable, and V_O_-ZnO seems less unstable than V_Zn_-ZnO. Hussain et al. [57] reached a similar conclusion by studying ZnO nano sheets. Therefore, the 4 × 4 × 1 supercell model was chosen for ZnO ML with Y and vacancy. The formation energy values of Y-doped ZnO ML with V_Zn_ or V_O_ were positive, indicating the instability of the systems. Figure 3a–d show the optimized structures of ZnO ML with V_Zn_, ZnO ML with V_O_, ZnO ML with Y and V_Zn_, and ZnO ML with Y and V_O_, called V_Zn_-ZnO, V_O_-ZnO, Y-V_Zn_-ZnO, and Y-V_O_-ZnO, respectively.

Figure 3a,b and Table 1 clearly show that the ZnO ML exhibited a distortion because of the vacancy. Compared with the Zn-O bond in ZnO ML, the Zn-O bond lengths adjacent to V_Zn_ in Figure 3a decreased to different degrees (as shown in Table 1), whereas the Zn-O bond lengths closest to V_O_ in Figure 3b increased slightly. The charge effect can be used to explain the changes in bond lengths and bond angles associated with the vacancies. A zinc atom is removed from the monolayer to form V_Zn_, and two negative charges are left in the monolayer. Therefore, other positively-charged zinc atoms would move toward V_Zn_. By contrast, the neighbouring oxygen atoms with negative charges would not be attracted by static electricity and would move further away. Therefore, the Zn-O bond lengths near the V_Zn_ would be shortened, explaining the changes in the bond angle. For V_Zn_, both zinc and oxygen moved closer to an oxygen site when the oxygen was removed [58]. In addition, the lattice parameters of ZnO ML, Y-ZnO, V_Zn_-ZnO, V_O_-ZnO, Y-V_Zn_-ZnO, and Y-V_O_-ZnO were 3.282, 3.329, 3.281, 3.282, 3.282, and 3.290 Å, respectively. The results indicate that the lattice constant of ZnO increased by Y doping, the lattice constant of ZnO decreased slightly by the presence of V_Zn_, and V_O_ had little effect on the lattice constant of ZnO. The ionic radius of Y^3+^ (0.9 Å) was larger than that of Zn^2+^ (0.74 Å), which increased the lattice parameter a of ZnO ML. The removal of the zinc and oxygen atoms resulted in a small change in the lattice parameters of ZnO ML.

### 3.2. Electronic Structure

#### 3.2.1. The Pristine ZnO ML and ZnO MLs with Y or Vacancy

**Figure 4:**
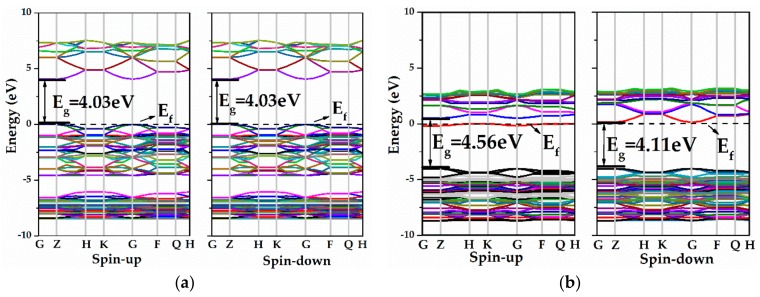

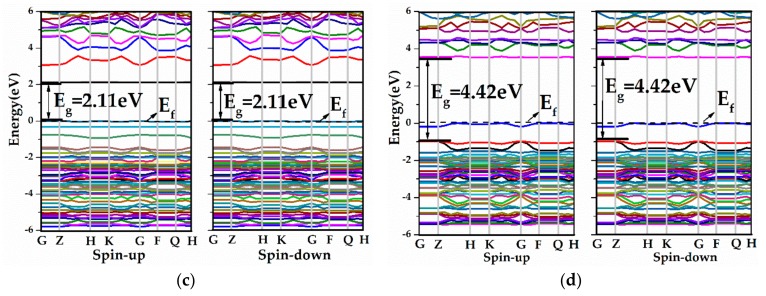
The band structures of (**a**) ZnO ML, (**b**) Y-ZnO, (**c**) V_Z__n_-ZnO, and (**d**) V_O_-ZnO.

The calculated band structures for pure ZnO ML and ZnO MLs with yttrium or vacancy (Y-ZnO, V_Zn_-ZnO, V_O_-ZnO) are presented in Figure 4. From Figure 4a,b, both valence band maximum (VBM) and conduction band minimum (CBM) were situated at the G point, indicating a direct bandgap. In Figure 4a, the bandgap of pure ZnO ML (4.03 eV) is greater than bulk ZnO (3.37 eV) [12], which can be explained by the quantum confinement effects, and it was confirmed theoretically and experimentally [59]. In addition, the energy band structures of the spin-up and spin-down components were symmetrical, indicating that the pristine ZnO ML is a non-magnetic semiconductor. From Figure 4b, Y-ZnO is a direct bandgap semiconductor with a bandwidth of 4.56 eV, which is larger than that of ZnO ML. It was noted that the Fermi level of Y-ZnO moved up and located approximately at the CBM, which produced a degenerate n-type semiconductor. This degenerate n-type semiconductor showed the Burstein–Moss effect [20]. A new impurity level appeared because of Y doping, and this impurity was located near the Fermi level in the energy band structure of the spin-up channel. The impurity level can serve as an effective recombination center and increases the separation rate of e^-^ and h^+^ when ZnO is illuminated by a light source. In addition, the energy band structures of the spin-up and spin-down channels were asymmetrical, implying that Y-ZnO is a magnetic semiconductor. Figure 4c clearly shows that the Fermi level of V_Zn_-ZnO moved slightly to the lower energy level, indicating that V_Zn_-ZnO is a degenerate p-type semiconductor, and shallow acceptor states were created around the Fermi level in the VBM, increasing the carrier concentration. For V_O_-ZnO, the forbidden bandgap increased, and the Fermi level shifted towards the higher energy region compared with the intrinsic ZnO ML, as shown in Figure 4d. Thus, the VB may be inferred to be completely occupied by electrons. On the other hand, the spin-up and spin-down components of V_Zn_-ZnO and V_O_-ZnO were symmetrical, indicating the absence of magnetism for the ZnO ML with vacancy.

**Figure 5:**
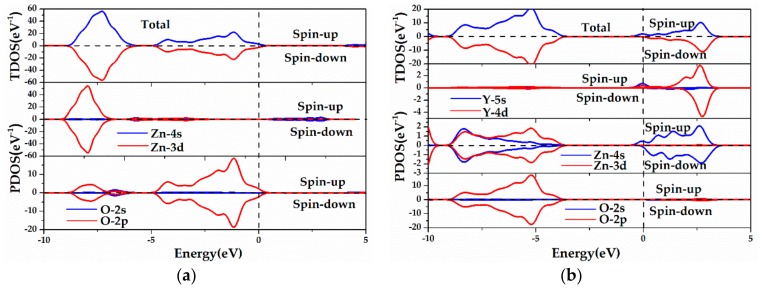

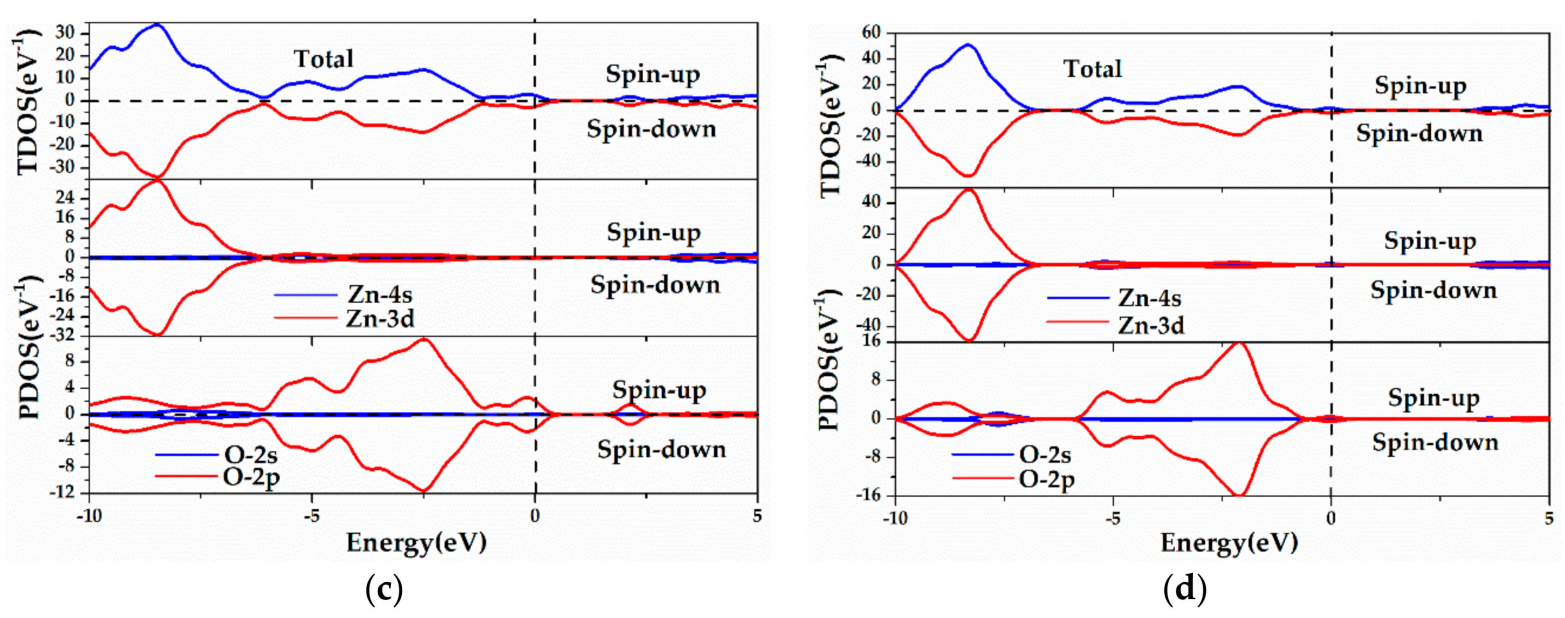
The calculated TDOS and PDOS (total and partial density of states) of (**a**) ZnO ML, (**b**) Y-ZnO, (**c**) V_Z__n_-ZnO, and (**d**) V_O_-ZnO.

Figure 5 plots the total and the partial density of states (TDOS, PDOS) of the pristine, Y single-doped ZnO MLs and ZnO MLs with vacancy, and these parameters were applied to analyze the distribution and occupancy of each related orbit of Zn, O, and Y elements. As shown in Figure 5a, the VB consisted of two parts: the lower part was composed of Zn-3d and O-2p states, whilst the upper part was mainly dominated by O-2p states. The hybridization of the Zn-3d and O-2p states occurred in the VB, and the bonding states were formed in this energy region [18]. The CB was mainly ascribed to Zn-4s and O-2p states. Therefore, the antibonding states in the CB of ZnO ML comprised Zn-4s and O-2p states [18]. From Figure 5b, the VB mainly consisted of O-2p and Zn-3d states, the VBM was determined by O-2p states, and the CB was mainly composed of Y-4d and Zn-4s states, with limited contribution from O-2p states. In addition, the chemical bonding of Zn, O, and Y atoms caused orbital hybridization between the Y atom and adjacent Zn and O atoms, and the electron density overlaps will increase with Y doping, which plays a crucial role in controlling the chemical properties of Y-ZnO. This process will also lead to the splitting of the donor energy level and expansion into an impurity band. Therefore, Y doping has a great impact on ZnO ML conductivity. In addition, the TDOS was asymmetrical, indicating that the spin-polarization mainly originated from the 4d electrons of the Y atom. For V_Zn_-ZnO and V_O_-ZnO, the bandgap of ZnO ML was mainly determined by Zn-4s states in the CB and O-2p states in the VB. For oxygen vacancy, the VBM and CBM moved to a lower energy region, the presence of V_O_ resulted in the splitting of the VB into two narrower bandgaps (−2 eV and 0 eV), as shown in Figure 5d. The upper energy level was located in the forbidden band, which is induced by oxygen vacancy [31]. The new energy level is beneficial to the transition of electrons under low-energy incident light. Moreover, vacancy could change the conductivity of ZnO ML by changing the carrier concentration near the Fermi level, which is similar to that of Y doping.

#### 3.2.2. Y-doped ZnO MLs with Vacancy

**Figure 6:**
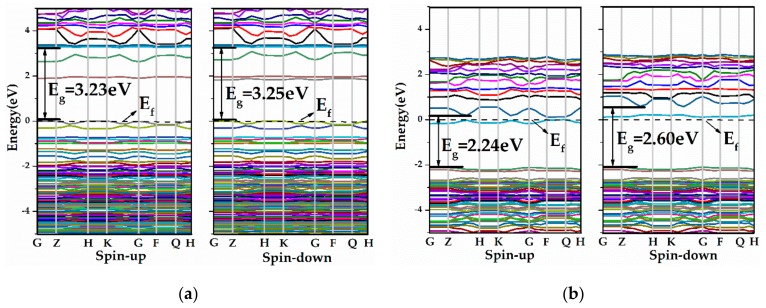
The band structures of (**a**)Y-V_Z__n_-ZnO, (**b**) Y-V_O_-ZnO.

The effects of V_Zn_ and V_O_ on the electronic structure of Y-doped ZnO ML were investigated. Figure 6 shows the energy band structures of ZnO MLs with Y and vacancy (V_Zn_ or V_O_), which were calculated by the GGA+U method. From Figure 6a, the VBM and CBM of Y-V_Zn_-ZnO were located at the G point, suggesting that Y-V_Zn_-ZnO is a direct bandgap semiconductor. The bandgap of Y-V_Zn_-ZnO was reduced to 3.23 eV, which is smaller than those of ZnO ML and Y-ZnO. Chemical bonds can be formed between a zinc atom and three adjacent oxygen atoms, so the V_Zn_ induced holes provided by three surrounding O atoms. The increase in hole concentration hindered the transition of electrons and the recombination of e^-^ and h^+^. In addition, the position of the Fermi level in Y-V_Zn_-ZnO was substantially consistent with that of ZnO ML, which was located near the VBM. From Figure 6b, the VBM of Y-V_O_-ZnO was situated at the G point, whereas the CBM was located between the Z and H points. Thus, Y-V_O_-ZnO is an indirect bandgap semiconductor. The bandgap of Y-V_O_-ZnO (2.24 eV) was smaller than that of Y-V_Zn_-ZnO. The Fermi level of Y-V_O_-ZnO lay near the CBM, which produced typical n-type metallic characteristics [23]. This means that shallow donor states were created near the CBM, thus leading to an increase in carrier concentration. Besides this, some new impurity levels were found near the CBM. The appearance of the impurity levels indicates that electrons were introduced, which is conducive to the separation of electron-hole pairs when the incident light is irradiated.

**Figure 7:**
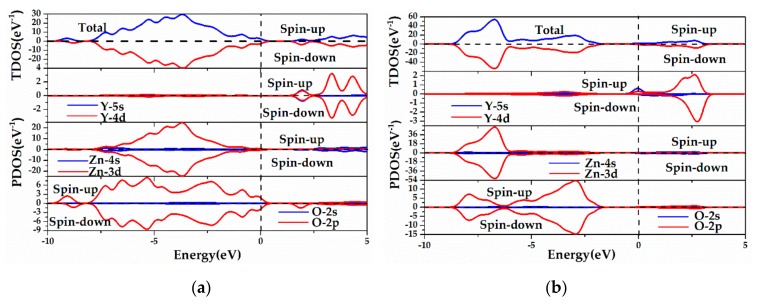
The calculated TDOS and PDOS of (**a**) Y-V_Z__n_-ZnO, (**b**) Y-V_O_-ZnO.

Figure 7 shows the TDOS and PDOS of ZnO MLs with Y and vacancy. Figure 7a shows that for Y-V_Zn_-ZnO, Y-4d states mainly acted at 2.00 eV, 3.40 eV, and 4.40 eV. Given the appearance of V_Zn_, the localization of O-2p states was weakened and the upper portion of the VB became diffuse. The incorporated Y atoms would have a stronger interaction with the O atoms, and orbital hybridization occurred near 2.0 eV between O-2p states and Y-4d states. Then, the impurity levels appeared. The spin-up and spin-down components of the Y-V_Zn_-ZnO DOS were almost symmetrical, indicating that the slight spin-polarization mainly came from the 2p electrons of O atoms. Figure 7b clearly shows that for Y-V_O_-ZnO, Y-4d states mainly acted at 2.70 eV, whereas Y-5s states mainly affected the DOS at −2.90 eV and 0 eV. The peak of the O-2p states moved to the lower energy region of −2.90 eV, which caused the movement of VBM. At the same time, the localization of the Zn-3d and O-2p states in VB became weak when V_O_ was in ZnO ML. The orbital hybridization occurred at −2.90 eV among Y-5s, Y-4d, and O-2p states. Moreover, the CBM dropped from 4.01 eV to 0.50 eV, which was induced by the action between Y-4d and Zn-4s states, resulting in a large reduction of the forbidden bandgap. The spin-up and spin-down components of the Y-V_O_-ZnO DOS were asymmetrical, suggesting that the spin-polarization mainly came from the 4d and 5s electrons of the Y atom. In summary, the forbidden band width of ZnO ML with Y and vacancy is reduced, and the introduced impurity level will act as a “springboard” for electrons to jump into the CB, which may result in a stronger optical absorption coefficient in the visible light region [60]. The value of DOS near the Fermi level increases because of Y doping, which is also advantageous to the transition of electrons.

#### 3.2.3. Population Analysis and Charge Density Difference

To understand the bonding between atoms, the Mulliken charges for ZnO ML were calculated. Table 2 shows the gains and losses of electrons of ZnO ML with and without Y or vacancy. The charge in ZnO ML doping systems was redistributed: Y and Zn atoms lost electrons, and the O atom obtained electrons. Hence, the Y atom incorporation will undergo a negative charge transfer with the adjacent Zn and O atoms. In addition, the charge change of Zn (1.07 e and 1.03 e) in V_Zn_-ZnO (Y-V_Zn_-ZnO) was slightly greater than that (0.98 e and 0.84 e) in V_O_-ZnO (Y-V_O_-ZnO), indicating that the covalent strength of the Zn-O bond in V_Zn_-ZnO (Y-V_Zn_-ZnO) was stronger than that in V_O_-ZnO (Y-V_O_-ZnO). For Y-doped ZnO ML with vacancy, the charge change of Y (1.58 e and 1.01 e) in Y-V_Zn_-ZnO (Y-V_O_-ZnO) was larger than that of Zn (1.03 e and 0.84 e), indicating that the Y-O bond in Y-V_Zn_-ZnO (Y-V_O_-ZnO) has a stronger covalent bond strength than Zn-O in Y-V_Zn_-ZnO (Y-V_O_-ZnO).

Table 3 shows the chemical bond lengths and bond populations of Y-doped ZnO MLs with and without vacancy. The bonding population indicates the degree of overlap of the two bonded atomic electron clouds, which can be used to obtain the properties of chemical bonds. The bond length of the Y-O bond revealed a linear relationship with the corresponding overlap population [61]. Table 3 shows that the degree of overlap of the electron cloud was inversely proportional to the bond length. The bond length of Y-O was larger than that of Zn-O (1.895 Å), because the radius of Y^3+^ (0.09 nm) is larger than that of Zn^2+^ (0.074 nm). In addition, the Y-O bond length of Y-V_Zn_-ZnO was shorter than that of Y-V_O_-ZnO, which indicates that the Zn and Y electron clouds were overlapping. Therefore, the transfer of electrons is more difficult for Y-V_O_-ZnO. The bond population of Y-O bond for Y-V_Zn_-ZnO was the largest, indicating the strongest overlap of electron clouds. Vacancy had little effect on the Y-O bond population of Y-V_O_-ZnO. In addition, the Zn-O bond lengths in Y-ZnO, Y-V_Zn_-ZnO, and Y-V_O_-ZnO were slightly larger than that in ZnO ML, but the bond population of Zn-O in Y-ZnO, and Y-V_Zn_-ZnO was slightly less than that in ZnO ML, indicating that the Zn-O bond in Y-ZnO and Y-V_Zn_-ZnO is more ionic than that in ZnO ML. The bond populations of Zn-O and Y-O in Y-ZnO were the same, indicating that the ionic strength of the Zn-O bond is similar to that of the Y-O bond. The Zn-O bond in Y-V_Zn_-ZnO was more ionic than the Y-O bond, but Y-V_O_-ZnO showed the opposite trend. The difference is due to the vacancy.

**Figure 8:**
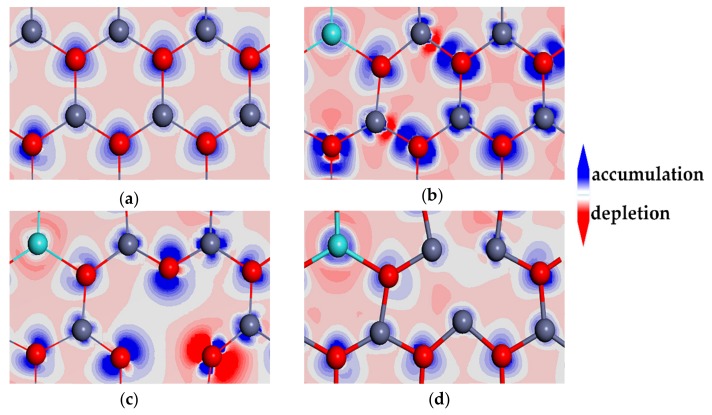
The charge density differences of (**a**) ZnO ML, (**b**) Y-ZnO, (**c**) Y-V_Zn_-ZnO, and (**d**) Y-V_O_-ZnO. Electron accumulation and electron depletion are shown in blue and red, respectively, and the areas with minimal change in the electron density are shown in white.

Figure 8 depicts the charge density differences of ZnO ML and Y-doped ZnO ML with or without vacancy. As shown in Figure 8b, the charge density distribution changed obviously with Y doping. The electron densities around Zn^2+^ (1.00 e) and Y^3+^ (1.14 e) ions are different, which can be seen from the Mulliken charge in Table 2. The incorporation of Y and vacancy into a crystal induced modifications of the electron density distribution in the space between crystal lattice ions, as provided in Figure 8c,d [61]. This result indicates that the covalent bond characteristic between Y and adjacent O atoms in Y-V_Zn_-ZnO was stronger than that in Y-ZnO and Y-V_O_-ZnO because of the overlap of electron clouds and a strong interaction. In addition, the electron cloud overlap of Zn-O bonds near the V_Zn_ in Figure 8c was significantly greater than that near the V_O_ in Figure 8d. The Zn-O bond near the V_Zn_ was more covalent, indicating that the electron transfer between the Zn atom and the adjacent O atom near the V_Zn_ was less than that near the V_O_.

### 3.3. Optical Properties

To better understand the application of Y-ZnO, V_Zn_-ZnO, V_O_-ZnO, Y-V_Zn_-ZnO, and Y-V_O_-ZnO in nanodevices, the optical properties should be calculated. In this work, the optical properties of ZnO ML with impurity or defect were obtained by the GGA+U method. In the linear response range, the macroscopic optical response function of the solid is described by the complex dielectric function of light *ε(ω)=ε_1_(ω)+iε_2_(ω)*. The dielectric function *ε(ω)* characterizes the linear response of the materials to electromagnetic radiation. The imaginary part *ε_2_(ω)* of the dielectric function is directly related to optical absorption coefficient, which can be calculated from the matrix element between the electronic wave functions of the occupied states and the unoccupied states according to the selection rule of the electronic transition [62]. The imaginary part *ε_2_(ω)* is expressed as follows [43]:

(2) $$\varepsilon_{2}\left(\omega \right)=\frac{{2e}^{2}\pi }{{\Omega \varepsilon }_{0}}\sum_{k,v,c}{\left|〈\varphi_{k}^{c}\left|u\cdot r\right|\varphi_{k}^{v}〉\right|}^{2}\delta \left(E_{k}^{c}{-E}_{k}^{v}-E\right),$$

where *ω* is the frequency of incident photons; *Ω* is the unit cell volume; $\varepsilon_{0}$ is the permittivity in free space; *k* is the reciprocal lattice vector; *v* and *c* stand for the VB and CB, respectively; *u* and *r* are the vector defining polarization of the incident electric field and the position vector; $\varphi_{k}^{c}$ and $\varphi_{k}^{v}$ are the wave functions of CB and VB, respectively [16].

The absorption coefficient *α(ω)* describes the percentage of light intensity attenuation at the unit distance of light wave propagation, which can be obtained from *ε_1_(ω)* and *ε_2_(ω)*, as follows [63]:

(3) $$\alpha \left(\omega \right)=\sqrt{2}\omega {\left[\sqrt{\varepsilon_{1}^{2}\left(\omega \right)+\varepsilon_{2}^{2}\left(\omega \right)}-\varepsilon_{1}\left(\omega \right)\right]}^{1/2}.$$

During the calculation of the imaginary part of the dielectric function and the absorption coefficient, the incident radiation is linearly polarized along the (100) direction. The energy range of the visible spectrum is 1.63–3.10 eV, and the corresponding wavelength is 760–400 nm.

**Figure 9:**
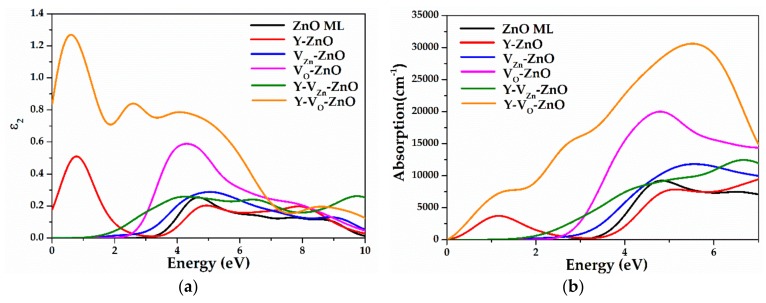
(**a**) The imaginary part of dielectric function and (**b**) the absorption coefficient of ZnO ML, Y-ZnO, V_Zn_-ZnO, V_O_-ZnO, Y-V_Zn_-ZnO, and Y-V_O_-ZnO.

Figure 9 depicts the results of imaginary part and the absorption coefficient of Y-doped ZnO MLs with vacancy. The calculation results of the pure ZnO ML and ZnO MLs with Y or vacancy are also shown for comparison. The peak was determined by the electron transition, which followed the selection rules. Figure 9a shows two distinct peaks in the dielectric function of ZnO ML, which are located at 4.70 eV and 7.70 eV, respectively. For Y-ZnO, the position of the absorption peak gradually shifted toward the higher energy region with Y doping, which is known as a blue-shift. A new peak appeared near 0.75 eV, which was mainly attributed to Y doping. For V_Zn_-ZnO, one main peak was shown at 4.70 eV and moved to 5.10 eV, which is called a blue-shift. For V_O_-ZnO, the main peak shifted to lower energy with the existence of V_O_, which is known as a red-shift. For Y-V_Zn_-ZnO, three main peaks were observed, and they exhibited a blue-shift. For Y-V_O_-ZnO, three main peaks showed a red-shift, and the values of the main peaks are larger than those of ZnO ML, Y-ZnO, V_Zn_-ZnO, V_O_-ZnO, and Y-V_Zn_-ZnO in the low energy region (< 7.0 eV). In Figure 9b, the peak of the absorption curve has a similar relative position to the imaginary part of the dielectric function. The absorption edge of ZnO ML was located at 4.0 eV, which is approximately equal to the bandgap of ZnO ML. For Y-ZnO, a new absorption peak emerged at 1.20 eV because Y doping induced impurity levels near the Fermi level. The formation of donor centers and the incorporation of Y^3+^ resulted in the formation of shallow levels or a sub band inside the forbidden band, resulting in a blue-shift approximately 4.90 eV in the absorption spectrum of Y-ZnO [25]. For vacancy, these results clearly show that the optical absorption coefficient of the ZnO ML is considerably affected by the presence of vacancy. For V_Zn_-ZnO, although the presence of V_Zn_ slightly enhanced the optical absorption properties of the ZnO ML in the visible light region (VIS), the optical absorption coefficient in ultraviolet (UV) region decreased at the same time. For V_O_-ZnO, the O-deficient ML showed an optical absorption band from 1.20 eV to 4.80 eV and the main optical absorption peak at 4.80 eV. The optical absorption coefficients in VIS and UV regions increased greatly, which indicates that the absorption coefficients in VIS and UV regions are enhanced by the presence of oxygen vacancy. For Y-V_Zn_-ZnO, in the range of 0-1.50 eV, the absorption coefficient was almost 0, which is smaller than that of Y-ZnO. The absorption edge of Y-V_Zn_-ZnO was red-shifted to the lower energy region near 2.0 eV, which is close to the energy difference from the VBM to the impurity levels. Given the presence of impurity levels, electrons were more likely to be excited from the VB to impurity levels and CB, which reduced the required electron energy, resulting in a red-shift at the edge of the absorption spectrum. The absorption coefficient of Y-V_Zn_-ZnO was larger than those of Y-ZnO and V_Zn_-ZnO in the VIS region. No new absorption peaks appeared in the visible light region for Y-V_Zn_-ZnO. The absorption coefficients of Y-V_Zn_-ZnO and Y-V_O_-ZnO were improved in the range from 2.0 eV to 4.0 eV, and the improvement is beneficial for the utilization of visible light. Given the appearance of V_Zn_, the interactions between Y and O atoms were enhanced, the number of free electrons in the system was reduced, and the electron transition was limited to a certain extent. Moreover, Y-V_O_-ZnO possessed the highest absorption coefficient in the VIS region compared with other doping systems, including Y-V_Zn_-ZnO and V_O_-ZnO. This is because the forbidden bandgap of Y-V_O_-ZnO decreased to 2.24 eV, and the Fermi level was located close to the CBM, which led to abundant electrons jumping from the VB to CB. In particular, the existence of V_O_ enhanced the interaction between Y and O atoms, thus decreasing the number of free electrons in the system and greatly improving the photocatalytic properties of ZnO ML. In general, compared with other doping systems, Y-V_O_-ZnO has the highest absorption coefficient and the strongest photocatalysis in the visible region.

### 3.4. Photocatalytic Reaction Mechanism and Installation Scheme of Photocatalytic Oxidation

**Figure 10:**
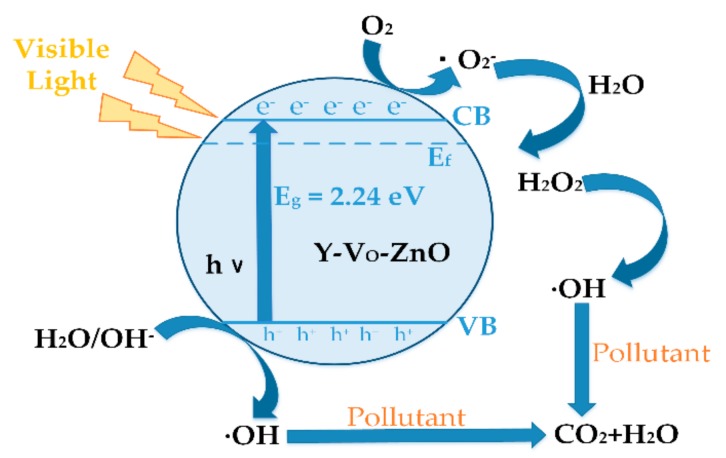
Photocatalytic mechanism of Y-V_O_-ZnO nanocatalyst.

Figure 10 shows the mechanism of the electron-hole pairs transfer in Y-V_O_-ZnO, leading to the catalytic degradation of pollutants under visible light irradiation. When Y-V_O_-ZnO is illuminated by sunlight, the electrons in the VB are excited by photons to jump to the CB, and the corresponding holes are retained in the VB. Photo-generated electrons and holes are separated by an electric field and move to the surface of semiconductor particles. The photo-generated pores have strong oxidizing properties and can oxidize substances adsorbed on the surface or solution of the semiconductor [2].

During the photocatalytic process, the hole in the VB diffuses to the photocatalyst surface and oxidises with H_2_O/OH^-^ to form hydroxyl radicals (·OH). The electrons in the CB move to the surface of the catalyst and react with O_2_ to produce superoxide radical anions (${\cdot O}_{2}^{-}$), which can be utilized as species for the degradation of organic pollutants by oxidation [64]. The surface reactions are given in Equations (4)–(9):

Y−V_O_−ZnO + hν (VIS light) → h^+^ + e^-^, (4)

H_2_O/OH^−^ + h^+^ → ·OH, (5)

O_2_ + e^−^ → O_−2_ (6)

(7) $$O_{2}^{-}+H_{2}O\to H_{2}O_{2}+O_{2}$$

H_2_O_2_ + e^−^ → ·OH, (8)

(9) $$h^{+}+O_{2}^{-}+\mathrm{OH}+\mathrm{Pollutant}\to H_{2}O+CO_{2}↑$$

From the above photocatalytic reaction processes, the photocatalytic property of Y-V_O_-ZnO was improved compared with the original ZnO ML. The doping of Y and vacancy can effectively promote the separation of electron-hole pairs, thus prolonging carrier lifetime. More carriers jump to the ZnO ML surface and participate in the photocatalytic reaction under visible light irradiation, which improves the redox reaction efficiency. Finally, the organic pollutants are decomposed into non-toxic H_2_O and CO_2_, achieving the goal of pollutant degradation.

**Figure 11:**
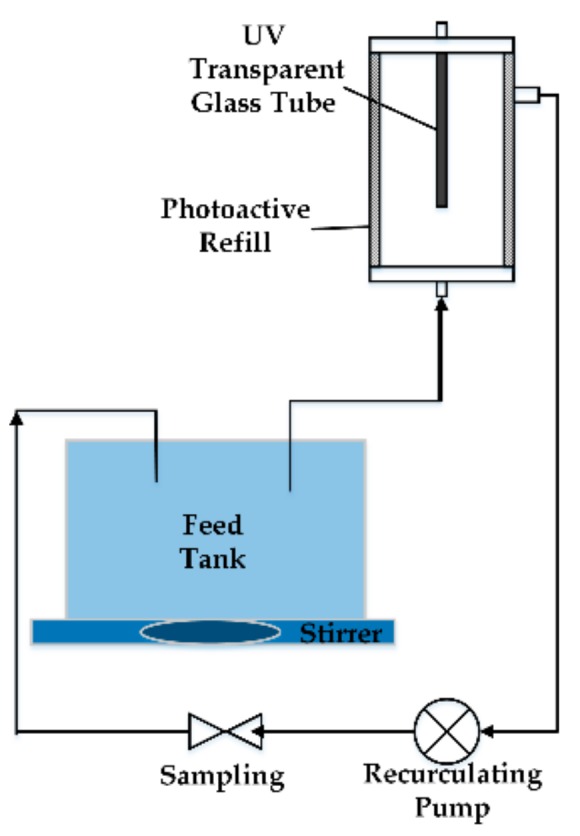
Schematic of solar catalyzed oxidation unit.

The photocatalytic oxidation of azo dye wastewater was performed in the equipment shown in Figure 11. The main component of the system was the flow reactor (Trojan Technologies, London, ON, Canada) with a refill covered with a titania–silica coating [65]. The solar reactor included one UV transparent glass tube as a receiver [66]. The photoactive refill was adhered to the inner walls of the reactor. The refill was composed of a thin Y-V_O_-ZnO layer mixed with a water solution of silicone binder. Before photocatalysis, the reactor was washed with excess immobilized photocatalyst to ensure that only a thin layer of the photocatalyst remained on the fabric. The azo dye wastewater was pumped from the feed tank into the reactor and circulated in the reactor. Samples were extracted termly for analysis until the azo dye wastewater was decomposed into a colorless, non-toxic liquid.

## 4. Conclusions

In summary, we explored crystal structure, structural stability, electronic structure, and optical properties of Y-doped ZnO ML with V_Zn_ or V_O_ by first-principles density functional theory. For comparison, a first-principles study of pure ZnO ML and ZnO MLs with Y or vacancy was calculated. The calculation results showed that the maximum formation energy value of Y-ZnO is −0.188 eV. The formation energies of ZnO MLs with vacancy and ZnO MLs with Y and vacancy (V_Zn_, V_O_) were positive, indicating that ZnO MLs with vacancy are unstable with or without Y. The bandgap of ZnO ML increased with the presence of Y. Meanwhile, impurity levels appeared near Fermi level in Y-ZnO, which was attributed to Y-4d and Zn-4s states. In addition, the bandgap of Y-doped ZnO ML with vacancy was smaller than those of ZnO ML, Y-ZnO, and V_O_-ZnO, which suggests that the emission of Y-doped ZnO MLs with V_Zn_ or V_O_ would exhibit a red-shift from 4.03 eV to 3.23 eV, 2.24 eV, respectively. The impurity level could be induced by the doping of Y or V_O_, which is advantageous for the electronic transition. Population analysis and charge density difference showed the change in the electrons and the bonding situation of Zn, O, and Y atoms in the ZnO monolayer doping systems. Moreover, the light absorptions of Y-doped ZnO MLs with vacancy were enhanced in the visible light region compared with that of the pristine ZnO ML and ZnO ML with one defect (Y, V_Zn_, or V_O_). In particular, the enhancement of the absorption coefficient of yttrium-doped ZnO ML in the VIS region became more pronounced with or without V_O_. This work can provide some references for the application of Y-V_O_-ZnO in the photocatalytic field.

## Figures and Tables

**Table 1:** Bond length *d* and bond angle *θ* of ZnO ML, V_Zn_-ZnO, and V_O_-ZnO (the atomic number is labeled in Figure 3).

Doping System	Bond	*d* (Å)	*Θ*	Value (°)
ZnO ML	Zn-O	1.895	Zn-O-Zn	120
			O-Zn-O	120
V_Zn_-ZnO	Zn_2_-O_Ⅳ_	1.884	O_Ⅳ_-Zn_2_-O_Ⅲ_	128.185
	Zn_2_-O_Ⅲ_	1.806	Zn_2_-O_Ⅲ_-Zn_4_	116.496
			Zn_2_-O_Ⅳ_-Zn_3_	132.858
V_O_-ZnO	Zn_2_-O_Ⅰ_	1.920	Zn_1_-O_Ⅰ_-Zn_2_	115.810
	Zn_1_-O_Ⅰ_	1.891	O_Ⅰ_-Zn_1_-O_Ⅱ_	113.397
			O_Ⅰ_-Zn_2_-O_Ⅲ_	115.772

**Table 2:** The atomic populations (Mulliken charge) of ZnO ML with Y, vacancy (V_Zn_, V_O_), and ZnO ML with Y and vacancy (V_Zn_, V_O_).

Doping System	Atomic Species	Mulliken Charge (e)			Total Charge (e)	Charge Change (e)
		s	p	d		
Y-ZnO	Zn	0.53	0.50	9.97	11.00	1.00
	O	1.84	5.14	0.00	6.99	−0.99
	Y	0.58	0.19	1.10	1.86	1.14
V_Zn_-ZnO	Zn	0.49	0.48	9.97	10.93	1.07
	O	1.85	5.15	0.00	7.00	−1.00
V_O_-ZnO	Zn	0.53	0.52	9.98	11.02	0.98
	O	1.84	5.20	0.00	7.04	−1.04
Y-V_Zn_-ZnO	Zn	0.51	0.49	9.97	10.97	1.03
	O	1.85	5.16	0.00	7.01	−1.01
	Y	0.26	0.09	1.06	1.42	1.58
Y-V_O_-ZnO	Zn	0.65	0.55	9.96	11.16	0.84
	O	1.86	5.05	0.00	6.91	−0.91
	Y	0.56	0.12	1.31	1.99	1.01

**Table 3:** Bond lengths (LB) and bond populations (PB) of Y-doped ZnO ML and Y-doped ZnO ML with V_Zn_ or V_O_.

Model	Y-O		Zn-O	
	LB (Å)	PB	LB (Å)	PB
ZnO ML	-	-	1.895	0.43
Y-ZnO	2.225	0.42	1.905	0.42
Y-V_Zn_-ZnO	2.204	0.50	1.900	0.42
Y-V_O_-ZnO	2.230	0.41	1.905	0.43

## References

[1] Wang C.L., Sun Z.X., Zheng Y., Hu Y.H. (2019). Recent progress in visible light photocatalytic conversion of carbon dioxide. J. Mater. Chem. A.

[2] Zhang F.B., Wang X.M., Liu H.N., Liu C.L., Wan Y., Long Y.Z., Cai Z.Y. (2019). Recent Advances and Applications of Semiconductor Photocatalytic Technology. Appl. Sci..

[3] Sun B., Yang X.P., Zhao D., Zhang L.Q. (2018). First-principles study of adsorption mechanism of NH_3_ on different ZnO surfaces on organics photocatalytic degradation purpose. Comput. Mater. Sci..

[4] Wang J.J., Yang X.T., Cao J.R., Wang Y., Li Q.X. (2018). Computational study of the electronic, optical and photocatalytic properties of single-layer hexagonal zinc chalcogenides. Comput. Mater. Sci..

[5] Beura R., Thangadurai P. (2017). Structural, optical and photocatalytic properties of graphene-ZnO nanocomposites for varied compositions. J. Phys. Chem. Solids.

[6] Fu S.Y., Chen J.R., Han H.S., Wang W.Z., Shi H.L., Fu J.L., Jia Y. (2019). ZnO@Au@Cu_2_O nanotube arrays as efficient visible-light-driven photoelectrod. J. Alloys Compd..

[7] Fujishima A., Honda K. (1972). Electrochemical Photolysis of Water at a Semiconductor Electrode. Nature.

[8] Chen S.F., Liu F.N., Xu M.Z., Yan J.F., Zhang F.C., Zhao W., Zhang Z.Y., Deng Z.H., Yun J.N., Chen R.Y. (2019). First-principles calculations and experimental investigation on SnO_2_@ZnO heterojunction photocatalyst with enhanced photocatalytic performance. J. Colloid Interface Sci..

[9] Tu N., Bui H.V., Trung D.Q., Duong A.T., Thuy D.M., Nguyen D.H., Nguyen K.T., Huy P.T. (2019). Surface oxygen vacancies of ZnO: A facile fabrication method and their contribution to the photoluminescence. J. Alloys Compd..

[10] Li X.Y., Liu D.X., Zhu B.Y., Wang J., Lang J.H. (2019). Facile preparation of ZnO/Ag_2_CO_3_ heterostructured nanorod arrays with improved photocatalytic activity. J. Phys. Chem. Solids.

[11] Singh G., Kumar S., Singh V.P., Vaish R. (2019). Transparent ZnO crystallized glass ceramics for photocatalytic and antibacterial applications. J. Appl. Phys..

[12] Mohamed W.S., Abu-Diefb A.M. (2018). Synthesis, characterization and photocatalysis enhancement of Eu_2_O_3_-ZnO mixed oxide nanoparticles. J. Phys. Chem. Solids.

[13] Zhang H.F., Tao Z., Xu W.G., Lu S.X., Yuan F. (2012). First-principles study of dopants and defects in S-doped ZnO and its effect on photocatalytic activity. Comput. Mater. Sci..

[14] Kumar M.R., Nagaswarupa H.P., Ravikumar C.R., Prashantha S.C., Nagabhushana H., Bhatt A.S. (2019). Green engineered nano MgO and ZnO doped with Sm^3+^: Synthesis and a comparison study on their characterization, PC activity and electrochemical properties. J. Phys. Chem. Solids.

[15] Cheng J.S., Wang P., Hua C., Yang Y.T., Zhang Z.Y. (2018). First principles investigations of the structural, electrical and optical properties of iron-doped zinc oxide (0 0 0 1) surfaces. Comput. Mater. Sci..

[16] Ma Z.H., Ren F.Z., Ming X.L., Long Y.Q., Volinsky A.A. (2019). Cu-Doped ZnO Electronic Structure and Optical Properties Studied by First-Principles Calculations and Experiments. Materials.

[17] El Hachimi A.G., NE M.L.O., El Yousfi A., Benyoussef A., El Kenz A. (2019). Enhancing optical absorption in visible light of ZnO co-doped with europium and promethium by first-principles study through modified Becke and Johnson potential scheme. J. Rare Earths.

[18] Jia X.F., Hou Q.Y., Xu Z.C., Qu L.F. (2018). Effect of Ce doping on the magnetic and optical properties of ZnO by the first principle. J. Magn. Magn. Mater..

[19] Sarfraz M., Ahmed N., Khizar U.H., Shahida S., Khan M.A. (2019). Structural optical and magnetic properties of transition metal doped ZnO magnetic nanoparticles synthesized by sol-gel auto-combustion method. Mater. Sci. Pol..

[20] Sun D., Tan C.L., Tian X.H., Huang Y.W. (2017). Comparative Study on ZnO Monolayer Doped with Al, Ga and in Atoms as Transparent Electrodes. Materials.

[21] Ivanova T., Harizanova A., Koutzarova T., Vertruyen B. (2017). Sol-gel derived ZnO:Y nanostructured films: Structural and optical study. Colloids Surf. A.

[22] Bazta O., Urbieta A., Piqueras J., Fernandez P., Addou M., Calvino J.J., Hungria A.B. (2019). Influence of yttrium doping on the structural, morphological and optical properties of nanostructured ZnO thin films grown by spray pyrolysis. Ceram. Int..

[23] Wang P., He J.F., Guo L.X., Yang Y.T., Zheng S.K. (2015). The electronic structures and optical properties of yttrium-doped zinc oxide with zinc interstitial defects calculated by first-principles. Mater. Sci. Semicond. Process..

[24] Lahmer M.A. (2018). The effect of doping with rare earth elements (Sc, Y, and La) on the stability, structural, electronic and photocatalytic properties of the O-terminated ZnO surface, A first-principles study. Appl. Surf. Sci..

[25] Qasim A.K., Jamil L.A., Chen Q. (2018). Enhanced Photoelectrochemical Water Splitting of Hydrothermally-Grown ZnO and Yttrium-doped ZnO NR Arrays. IOP Conf. Ser. Mater. Sci. Eng...

[26] Hashmi J.Z., Siraj K., Naseem S., Shaukat S. (2016). Dopant-induced modifications in structural and optical properties of ZnO thin films prepared by PLD. Mater. Res. Express.

[27] Bai L.N., Sun H.M., Lian J.S., Jiang Q. (2012). Tunable UV Absorption and Mobility of Yttrium-Doped ZnO using First-Principles Calculations. Chin. Phys. Lett..

[28] Tang Y.W., Zhou H., Zhang K., Ding J., Fan T.X., Zhang D. (2015). Visible-light-active ZnO via oxygen vacancy manipulation for efficient formaldehyde photodegradation. Chem. Eng. J..

[29] Wang Z.L., Su S.C., Younas M., Ling F.C.C., Anwand W., Wagner A. (2015). The Zn-vacancy related green luminescence and donor-acceptor pair emission in ZnO grown by pulsed laser deposition. RSC Adv..

[30] Lahmer M.A. (2016). The effect of growth conditions and vacancies on the electronic, optical and photocatalytic properties of the ZnO (1010) surface. Mater. Chem. Phys..

[31] Bai L.L., Lin Z.P., Wen M.R., Dong H.F., Liu Z.T., Chen S.S., Wu F.G. (2019). Vacancies inducing electronic and optical properties in 2D ZnO:Be/Mg. Physica B.

[32] Catellani A., Ruini A., Calzolari A. (2015). Optoelectronic properties and color chemistry of native point defects in Al:ZnO transparent conductive oxide. J. Mater. Chem. C.

[33] Meng Z.S., Mo X.M., Cheng X., Zhou Y.L., Tao X.M., Ouyang Y.F. (2017). Interactions between Er dopant and intrinsic point defects of ZnO: A first-principles study. Mater. Res. Express.

[34] Qu L.F., Hou Q.Y., Jia X.F., Xu Z.C., Zhao C.W. (2018). Effects of Eu doping and O vacancy on the magnetic and optical properties of ZnO. Physica B.

[35] Li W.L., Hou Q.Y., Xu Z.C., Zhao C.W. (2019). Study of point defect on the stability and magneto-optical properties of ZnO:Cu by first-principles. Mol. Phys..

[36] Li C., Hou Q.Y. (2018). Effects of Y doping with point defects on the ferromagnetic properties of ZnO(0001)-Zn polar surface. Appl. Surf. Sci..

[37] Zhang L.L., Zhu D., He H.X., Wang Q., Xing L.L., Xue X.Y. (2017). Enhanced piezo/solar-photocatalytic activity of Ag/ZnO nanotetrapods arising from the coupling of surface plasmon resonance and piezophototronic effect. J. Phys. Chem. Solids.

[38] Chen H.F., Tan C.L., Zhang K., Zhao W.B., Tian X.H., Huang Y.W. (2019). Enhanced photocatalytic performance of ZnO monolayer for water splitting via biaxial strain and external electric field. Appl. Surf. Sci..

[39] Freeman C.L., Claeyssens F., Allan N.L., Harding J.H. (2006). Graphitic Nanofilms as Precursors to Wurtzite Films: Theory. Phys. Rev. Lett..

[40] Claeyssens F., Freeman C.L., Allan N.L., Sun Y., Ashfold M.N.R., Harding J.H. (2005). Growth of ZnO thin films—Experiment and theory. J. Mater. Chem..

[41] Deng X.Y., Yao K., Sun K.J., Li W.X., Lee J., Matranga C. (2013). Growth of Single- and Bilayer ZnO on Au(111) and Interaction with Copper. J. Phys. Chem. C.

[42] Tan C.L., Xu D.S., Zhang K., Tian X.H., Cai W. (2015). Electronic and Magnetic Properties of Rare-Earth Metals Doped ZnO Monolayer. J. Nanomater..

[43] Wang Q.B., Zhou C., Wu J., Lu T., He K.H. (2015). GGA+U study of the electronic and optical properties of hexagonal BN phase ZnO under pressure. Comput. Mater. Sci..

[44] Perdew J.P., Chevary J.A., Vosko S.H., Jackson K.A., Pederson M.R., Singh D.J., Fiolhais C. (1992). Atoms, molecules, solids, and surfaces: Applications of the generalized gradient approximation for exchange and correlation. Phys. Rev. B.

[45] Perdew J.P., Burke K., Ernzerhof M. (1996). Generalized Gradient Approximation Made Simple. Phys. Rev. Lett..

[46] Hammer B., Hansen L.B., Norskov J.K. (1999). Improved adsorption energetics within density-functional theory using revised Perdew-Burke-Ernzerhof functionals. Phys. Rev. B.

[47] Wen J.Q., Zhang J.M., Chen G.X., Wu H., Yang X. (2018). The structural, electronic and optical properties of Nd doped ZnO using first-principles calculations. Physica E.

[48] Vanderbilt D. (1990). Soft self-consistent pseudopotentials in a generalized eigenvalue formalism. Phys. Rev. B.

[49] Monkhorst H.J. (1976). Special points for Brillouin-zone integrations. Phys. Rev. B.

[50] Majid A., Akram W., Dar A. (2014). DFT study of electronic and structural properties of Sm:GaN. Comput. Mater. Sci..

[51] Hou Q.Y., Ji X.F., Xu Z.C., Zhao C.W., Qu L.F. (2018). Effects of Li doping and point defect on the magnetism of ZnO. Ceram. Int..

[52] Tan C.L., Sun D., Tian X.H., Huang Y.W. (2016). First-Principles Investigation of Phase Stability, Electronic Structure and Optical Properties of MgZnO Monolayer. Materials.

[53] Ren J., Zhang H., Cheng X.L. (2013). Electronic and Magnetic Properties of all 3d Transition-metal Doped ZnO Monolayers. Int. J. Quantum Chem..

[54] Chen L.L., Wang A.P., Xiong Z.H., Shi S.Q., Gao Y.F. (2019). Effect of hole doping and strain modulations on electronic structure and magnetic properties in ZnO monolayer. Appl. Surf. Sci..

[55] Peng Q., Liang C., Ji W., De S. (2013). A first principles investigation of the mechanical properties of g-ZnO: The graphene-like hexagonal zinc oxide monolayer. Comput. Mater. Sci..

[56] Mendoza-Estrada V., Gonzalez-Garcia A., Barragan-Yani D., Lopez-Perez W., Rivera-Julio J., Gonzalez-Hernandez R. (2017). Ferromagnetic orderings in Co*_x_*Cu*_y_*Zn_1__−__(_*_x_*_+_*_y_*_)_O by GGA and GGA+U formalisms within density functional theory. Comput. Mater. Sci..

[57] Hussain T., Kaewmaraya T., Khan M., Chakraborty S., Islam M.S., Amornkitbamrung V., Ahuja R. (2017). Improved sensing characteristics of methane over ZnO nano sheets upon implanting defects and foreign atoms substitution. Nanotechnology.

[58] Kohan A.F., Ceder G., Morgan D. (2000). First-principles study of native point defects in ZnO. Phys. Rev. B.

[59] Sun Z.Q., Liao T., Dou Y.H., Hwang S.M., Park M.S., Jiang L., Kim J.H., Dou S.X. (2014). Generalized self-assembly of scalable two-dimensional transition metal oxide nanosheets. Nat. Commun..

[60] Cheng J.S., Wang P., Hua C., Yang Y.T., Zhang Z.Y. (2018). The Impact of Iron Adsorption on the Electronic and Photocatalytic Properties of the Zinc Oxide (0001) Surface: A First-Principles Study. Materials.

[61] Zheng H.Y., Li J., Zhang X.C., Li Z., Xie K.C. (2015). Structural and electronic properties of Cu-doped Zn_5_(OH)_6_(CO_3_)_2_ from first principles. J. Mater. Sci..

[62] Luan Z.H., Sun D., Tan C.L., Tian X.H., Huang Y.W. (2017). First-principles calculations of electronic structure and optical properties of Be-doped ZnO monolayer. Integr. Ferroelectr..

[63] Saha S., Sinha T.P., Mookerjee A. (2000). Electronic structure, chemical bonding, and optical properties of paraelectric BaTiO_3_. Phys. Rev. B.

[64] Pawar R.C., Choi D.H., Lee J.S., Lee C.S. (2015). Formation of polar surfaces in microstructured ZnO by doping with Cu and applications in photocatalysis using visible light. Mater. Chem. Phys..

[65] Grzechulska-Damszel J., Morawski A.W. (2009). Water Purification Using a Novel Reactor with Photoactive Refill. Catal. Lett..

[66] Jamil T.S., Ghaly M.Y., Fathy N.A., Abd el-halim T.A., Österlund L. (2012). Enhancement of TiO_2_ behavior on photocatalytic oxidation of MO dye using TiO_2_/AC under visible irradiation and sunlight radiation. Sep. Purif. Technol..

